# Impact of COVID‐19 policies on perceptions of loneliness in people aged 75 years and over in the cognitive function and aging study (CFAS II)

**DOI:** 10.1111/jgs.18099

**Published:** 2022-11-12

**Authors:** Connor D. Richardson, Hannah Roscoe, Emma Green, Racheal Brooks, Linda Barnes, Fiona E. Matthews, Carol Brayne

**Affiliations:** ^1^ Population Health Sciences Institute Newcastle University, Newcastle Biomedical Research Building, Campus for Ageing and Vitality Newcastle upon Tyne UK; ^2^ St Helens and Knowsley Teaching Hospitals NHS Trust St Helens, Merseyside UK; ^3^ University of Cambridge, Department of Public Health & Primary Care Strangeways Research Laboratory, Worts Causeway Cambridge UK

**Keywords:** COVID‐19, Coronavirus, Epidemiology, Loneliness

## Abstract

**Background:**

The COVID‐19 pandemic and associated social distancing measures have profoundly impacted society and social contact patterns, with older people disproportionately affected. Concerns have been raised about a resulting pandemic of loneliness in older people, although the current evidence is mixed. This study provides a unique perspective on the prevalence of loneliness in a population cohort of older people before the pandemic, followed up after the introduction of social restrictions.

**Methods:**

Data analysis was conducted using Wave 3 of the longitudinal Cognitive Function and Aging Study II (2018–2019) and a sub‐study focusing on experiences during the COVID‐19 pandemic (2020). The sample comprised 379 adults aged over 75 living in Cambridge, Newcastle, and Nottingham. Multivariable binary logistic regression was conducted to identify correlates of prevalent loneliness, adjusted for confounding covariates, during the pandemic. The prevalence of loneliness during the pandemic was compared to loneliness in 2018–2019.

**Results:**

Prevalence of loneliness in this sample during the pandemic was 25.1% (95% CI 20.9%–29.7%) compared to 17.2% (95% CI 13.7%–21.3%) in 2018–2019 (χ^2^ *=* 14.1, *p* < 0.01). Variables associated with increased odds of prevalent loneliness included: prior loneliness, living alone, female gender, living in an area of higher deprivation, frequent pre‐pandemic social contact at community groups, and separation from family during the pandemic, adjusted for age and sex. Weekly technology‐mediated contact using telephone or video calls was associated with lower odds of loneliness.

**Conclusions:**

COVID‐19 recovery plans should address loneliness in older people. Target groups should include those who have previously been lonely, people who live alone, those living in deprived areas, and those who had previously been socially active through community groups.

**Key points**

- Loneliness in older people increased from 17.2% to 25.1% since COVID‐19 social distancing restrictions came into place.
- Prior loneliness, living alone, female gender, living in an area of higher deprivation, frequent pre‐pandemic social contact at community groups, separation from family during the pandemic increased the risk of loneliness.
- Technology facilitated contact was associated with lower odds of loneliness.

**Why does this paper matter?**

This longitudinal study of 379 community dwelling older adults reveals the pandemic is associated with increased prevalence of loneliness in older people. COVID‐19 recovery plans should address loneliness in older people. Target groups should include those who were lonely before the pandemic, people living alone, those in deprived areas, and those who had previously been socially active through community groups.

## INTRODUCTION

Loneliness is a subjective negative experience arising from a perceived deficit in the quality or quantity of social relationships.^1^, ^2^, ^3^, ^4^ Loneliness among older people is a recognized public health concern and is associated with many health risks, including increased risk of mortality, frailty, and functional decline.^5^, ^6^, ^7^ The risk of loneliness particularly increases in the ‘oldest‐old’ age groups, typically 80 or 85 and above.^8^

It is difficult to determine precisely the prevalence of loneliness in older people. Narrative reviews of evidence from Europe and North America have reported estimated rates of any loneliness in older adults of around a third,^9^, ^10^ with being ‘always or often’ lonely fairly consistent at around 8%–10%.^11^ One narrative review found rates of loneliness of 20%–30% in the 60–79‐year‐old age group and 40%–50% in those over 80.^8^

The COVID‐19 pandemic has caused over 118 million infections and 4 million deaths worldwide.^12^ The pandemic had profound impacts on societies, with many countries introducing unprecedented restrictions on the movement of people and freedom of association to slow the spread of the virus,^13^, ^14^ and disruption and or closure of usual health and social care services.^15^, ^16^ Older people have been disproportionately affected by the most stringent restrictions. In England, of those identified as clinically extremely vulnerable and advised to ‘shield’ at home, 63% were aged over 60, and 43% over 70.^17^ Therefore, it has been hypothesized that older people may be particularly affected by some of the indirect consequences of the pandemic, with increases in loneliness a particular concern.^18^, ^19^ Indeed, some have predicted a ‘loneliness pandemic’^20^, ^21^ among older people, arising from increased social isolation and the perceived threat of the virus.^22^ Given the link between loneliness and a range of health problems, there are concerns that increased loneliness related to the pandemic will lead to poorer health in this age group.^18^

Studies have suggested multiple theories on how the pandemic has affected the experience of loneliness in older adults. There are particular concerns for disadvantaged groups with prevalent loneliness before the pandemic becoming lonelier due to the implementation of social distancing restrictions.^18^, ^21^ There is also the potential for older people more generally who had frequent social contact, and were not lonely, before the pandemic becoming lonely due to disruption of their social networks.^23^ It has been suggested that those most at risk of loneliness are older people whose social contact relied on leaving the home such as: attending community or religious groups which restrictions largely curtailed.^18^, ^21^ This has not yet been explored empirically.

There is conflicting evidence for the ‘epidemic of loneliness’ in older people. Population‐level data in the UK indicate that increases in loneliness are highest in areas with high concentrations of younger people.^24^ Epidemiological studies in the UK, US, Hong Kong, Chile, and Europe have found mixed results, with some showing increased prevalence of loneliness in older people,^22^, ^25^, ^26^, ^27^, ^28^, ^29^ while others show no significant change from before the pandemic.^30^, ^31^, ^32^, ^33^ Qualitative research has also highlighted resilience shown by older people during the pandemic, with older adults deploying a range of coping strategies, including staying busy, pursuing hobbies, and undertaking exercise.^23^, ^34^, ^35^ Changing patterns of technology use has also been highlighted as a coping strategy.^23^, ^34^

This paper analyses prospective observational data on loneliness in adults aged over 75 years living in three areas of England and already part of an ongoing longitudinal study. It compares data collected during the COVID‐19 pandemic to data from the same individuals in the preceding 2 years. It investigates the prevalence of loneliness during the pandemic and the risk and protective factors for loneliness. It adds to the current literature by focusing on the 75 and over age group, including pre‐pandemic social contact and technology‐mediated contact as a potential risk and protective factors.

## METHODS

### Data source

This study is an analysis of observational data from the second Cognitive Function and Aging Study (CFAS II),^36^ and a nested study on experiences of older people during the pandemic – the ‘Social impacts of COVID‐19 policies for older people’ (OPPO) study.

#### Cognitive function and aging study II (CFAS II)

CFAS II is a population‐representative longitudinal study examining older adults' health, wellbeing, and cognition living in community settings in three English study centers: Cambridgeshire, Newcastle and Nottingham, recruited in 2008–2011. A population‐representative sample was drawn from individuals aged 65 and above registered with a general practice within the boundaries of the study centers.^37^ Since initial recruitment, there have been three primary data collection waves. All data collection waves have been drawn from the initial population‐representative sample. There has been attrition at each wave as participants have died or declined to participate (Figure S1).^37^

### CFAS II Wave 3

The CFAS II Wave 3 data were gathered 10 years after Wave 1 as part of a feasibility risk reduction trial funded by Alzheimer's Research UK (ARUK). The trial aimed to investigate the feasibility of an internet‐based intervention for dementia risk reduction, a platform based on the HATICE study previously tested in continental Europe.^38^ CFAS II cohort members who had completed CFAS II Waves 1 and 2 were eligible for the trial. These individuals were then screened against the following criteria: no previous indications of memory issues (no diagnosis of dementia and their last Mini‐Mental State Examination score was >21); aged 75–89; and cardiovascular risk score >1 based on a range of factors including hypertension, angina, smoking status, and physical inactivity. Full information on the entire CFAS II data flow (Figure S1).

### Social impacts of COVID‐19 policies for older people (OPPO)

**FIGURE 1:**
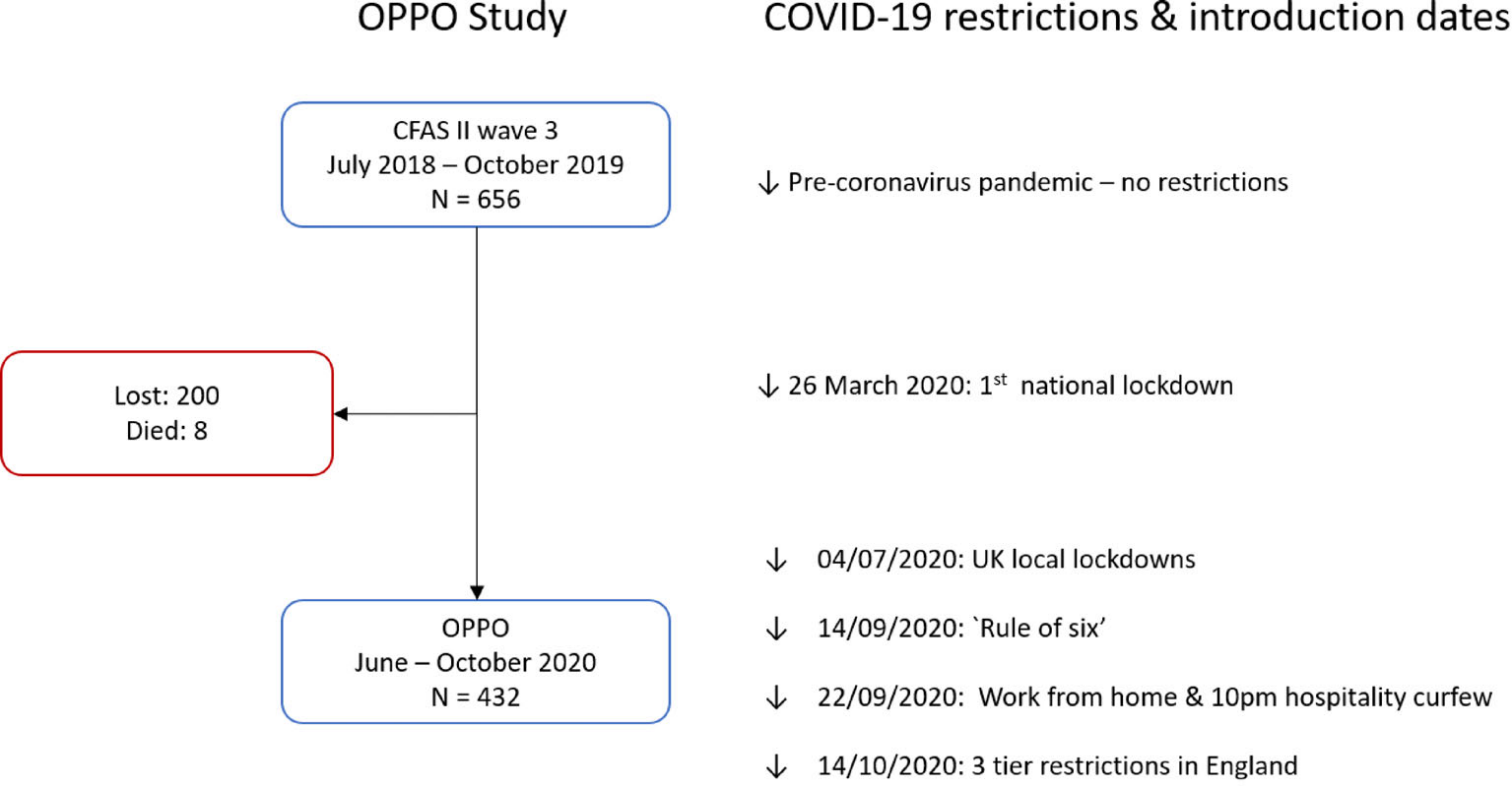
Data flow chart of CFAS II wave 3 and OPPO study with COVID‐19 social distancing restrictions and introduction dates. Additional information on timeline of COVID‐19 restrictions & OPPO data collection (appendix figures 2 & 3).

Following the onset of the COVID‐19 pandemic, an application was made to ARUK to use existing funded staff to conduct a nested study on the impacts COVID‐19 policies. This study aimed to explore the impact of the pandemic and COVID‐19 restriction measures on the cohort, including loneliness, social networks, and wellbeing. All participants who had participated in the recent CFAS II Wave 3 feasibility trial baseline and follow‐up were approached to participate, excluding those who had died as notified by NHS Digital (Figure 1). The data collection instrument used was an adaptation of a questionnaire on older people's experiences of COVID‐19 developed by the Canadian Longitudinal Study on Aging^39^ and also included questions from previous CFAS II waves, allowing for comparisons to be made across time. A further application was made to UK Research Institute (UKRI) with the Economic and Social Research Council (ESRC) for funds to expand the OPPO study and conduct a second wave of OPPO interviews; allowing the research team to assess these changes over the course of the pandemic. (Full information on entire CFAS II data flow in Data S1).

### Perceived loneliness

The outcome of interest was subjective loneliness. In CFAS II Wave 3, this was measured using the direct loneliness measure: ‘Do you feel lonely?’ This type of direct measure is widely used in previous studies with older people,^1^, ^2^, ^3^, ^4^ and has been shown to be well understood by this group.^40^, ^41^ The Office for National Statistics (ONS) also recommends that, if only one measure of loneliness is to be used, a direct rather than indirect measure is preferable.^42^ In the OPPO study, loneliness was measured using the loneliness item of the 10‐item Centre for the Epidemiological Studies of Depression Short Form (CES‐D‐10) scale. One of the items is a direct loneliness question ‘In the past week, how often did you feel lonely? The CES‐D‐10 has been shown to have validity in the older population,^43^ and the loneliness item has good concurrent validity with other ‘gold standard’ measures of loneliness, such as the UCLA indirect loneliness scale.^44^ Both loneliness variables were dichotomized for analysis (Table 2).

### Predictors of perceived loneliness

Study center, gender, and years in full‐time education were recorded as part of the administrative data at Wave 1. Deprivation based on the individual's home address was measured using the Townsend Deprivation Index, a validated measure of material deprivation of a geographical area calculated by combining Census data on unemployment, non‐car ownership, non‐home ownership, and overcrowding.^45^ Age, living alone or cohabiting and self‐rated health for age were analyzed. Self‐rated health was measured by asking participants to rate their health for someone of their age as ‘Excellent’, ‘Good’, ‘Fair’ or ‘Poor’. For analyses, these were recoded as ‘Excellent/Good’ and ‘Fair/Poor’. Measures of social contact were selected based on the theoretical literature on social contact loneliness^41^, ^46^ and previous studies that have operationalized social contact as a potential predictor of loneliness.^47^

### Pre‐pandemic social contact (CFAS II Wave 3)

Pre‐pandemic social contact was assessed using two questions asked in CFAS II Wave 3:

1. Frequency of contact with family and friends (Monthly or less, at least weekly, daily)
2. Frequency of contact through community groups (No, Yes less than weekly, yes more than weekly).
3. Contact with relatives either over the phone or using Skype or other similar face to face technologies? (Daily, 2–3 times a week, at least weekly, monthly, less often).

### Pre‐pandemic loneliness

In line with other similar studies,^48^ pre‐pandemic levels of loneliness as assessed in Wave 3 using the question ‘Do you feel lonely?’ (No, Infrequently, Frequently/Persistently).

### During‐pandemic social contact (OPPO study)

Levels of social contact were assessed using three questions assessing contact with friends and family. These were:

1. Have you experienced separation from friends and family? (No, Yes)
2. Have you left the house in the last month to meet friends or relatives? Responses (No, Yes)
3. Have you been under self‐quarantine in the past month, which means that you have only had contact with your immediate household members? (No, Yes)
4. Contact with children or other relatives either over the phone or using Skype or other similar face to face technologies? (Daily, 2–3 times a week, at least weekly, at least monthly, less often)

### Statistical analysis

Three hundred seventy‐nine had complete data for all variables of interest, and the remaining 38 were coded as ‘Missing’ and removed from the dataset.

Risk and protective factors for loneliness during the pandemic were selected using a covariate selection procedure and analyzed by binary logistic regression on the dichotomous measure of prevalent loneliness. Variables that were not significantly associated with loneliness in bivariate analyses were not included in the final analysis.

Proportions of lonely and non‐lonely individuals were calculated using the dichotomized pre‐ and during‐pandemic loneliness measures and assessed graphically. Significance testing of the difference in proportions of lonely versus non‐lonely individuals pre‐ compared to during the pandemic was conducted using McNemar's test for paired categorical data, with significance set at *p* < 0.05.

## RESULTS

Data from CFAS II Wave 3 and the OPPO study were available for 417 individuals. Table 1 shows the characteristics of the study sample and missing data.

**TABLE 1:** Sample characteristics, *p* values determined from chi‐square test.

		Included sample						Missing data		
		Total (*n* = 379)		Men (*n* = 196, 51.7%)		Women (*n* = 183, 48.3%)		Missing (*n* = 38, 9.1%)		
		*N*	%	*N*	%	*N*	%	*N*	%	*p*
Centre	Cambridge	162	*42.7*	91	*46.6*	71	*38.8*	21	*55.3*	0.1
	Newcastle	119	*31.4*	52	*26.5*	67	*36.6*	6	*15.8*	
	Nottingham	98	*25.9*	53	*27*	45	*24.6*	11	*29*	
Sex	Men	196	*51.7*	‐	*‐*	‐	*‐*	14	*36.8*	**<0.1**
	Women	183	*48.3*	‐	*‐*	‐	*‐*	24	*63.2*	
Age group	75–79	125	*33*	63	*32.1*	62	*33.9*	15	*39.5*	**<0.1**
	80–84	150	*39.6*	79	*40.3*	71	*38.8*	8	*21.1*	
	85+	104	*27.4*	54	*27.6*	50	*27.3*	15	*39.5*	
Race	White	375	*98.9*	194	98.9	181	*99*	38	*100*	0.9
	Non‐White	4	*1.1*	2	*1.1*	2	*1*	0	*0*	
Marital status	Married/cohabiting	232	*61.2*	154	*78.6*	78	*42.6*	18	*47.4*	**0.09**
	Widowed	117	*30.9*	26	*13.3*	91	*49.7*	19	*50*	
	Divorced	19	*5*	9	*4.6*	10	*5.5*	1	*2.6*	
	Single	11	*2.9*	7	*3.6*	4	*2.2*	0	*0*	
Accommodation	House/flat	361	*95.3*	191	*97.5*	170	*92.9*	30	*79*	**<0.1**
	Warden controlled flat	16	*4.2*	4	*2*	12	*6.6*	1	*2.6*	
	Granny flat	2	*0.5*	1	*0.6*	1	*0.5*	2	*5.3*	
	Private/charity nursing	0	*0*	0	*0*	0	*0*	1	*2.6*	
Living alone?	No	232	*61.2*	152	*77.6*	80	*43.7*	22	*57.9*	0.7
	Yes	147	*38.8*	44	*22.5*	103	*56.3*	16	*42.1*	
Deprivation	1st quintile (least deprived)	136	*35.9*	73	*37.2*	63	*34.4*	12	*31.6*	0.6
	2nd quintile	115	*30.3*	63	*32.1*	52	*28.4*	15	*39.5*	
	3rd quintile	55	*14.5*	26	*13.3*	29	*15.9*	3	*7.9*	
	4th quintile	44	*11.6*	24	*12.2*	20	*10.9*	4	*10.5*	
	5th quintile (most deprived)	29	*7.7*	10	*5.1*	19	*10.4*	4	*10.5*	
Years in education	0–9	39	*10.3*	15	*7.7*	24	*13.1*	6	*15.8*	0.6
	10	159	*42*	88	*44.9*	71	*38.8*	14	*36.8*	
	10+	181	*47.8*	93	*47.5*	88	*48.1*	18	*47.4*	
Self‐assessed health for age	Excellent	54	*14.3*	32	*16.3*	22	*12*	6	*16.7*	0.9
	Good	217	*57.3*	118	*60.2*	99	*54.1*	21	*58.3*	
	Fair	85	*22.4*	33	*16.8*	52	*28.4*	8	*22.2*	
	Poor	23	*6.1*	13	*6.6*	10	*5.5*	1	*2.8*	
Interview date	Period 1: 8 June–3 July	50	*13.2*	21	*11.5*	29	*14.8*	7	*18.4*	0.7
	Period 2: 4 July–31 July	100	*26.4*	47	*25.7*	53	*27*	7	*18.4*	
	Period 3: 1 August–13 September	108	*28.5*	54	*29.5*	54	*27.6*	11	*29*	
	Period 4: 14 September–26 October	121	*31.9*	61	*33.3*	60	*30.6*	13	*34.2*	

*Note*: Significance of bold values in Sex: p = 0.08, Significance of bold values in Age group: p = 0.07, Significance of bold values in Acommodation: *p* = < 0.01.

### Prevalence of perceived loneliness

**FIGURE 2:**
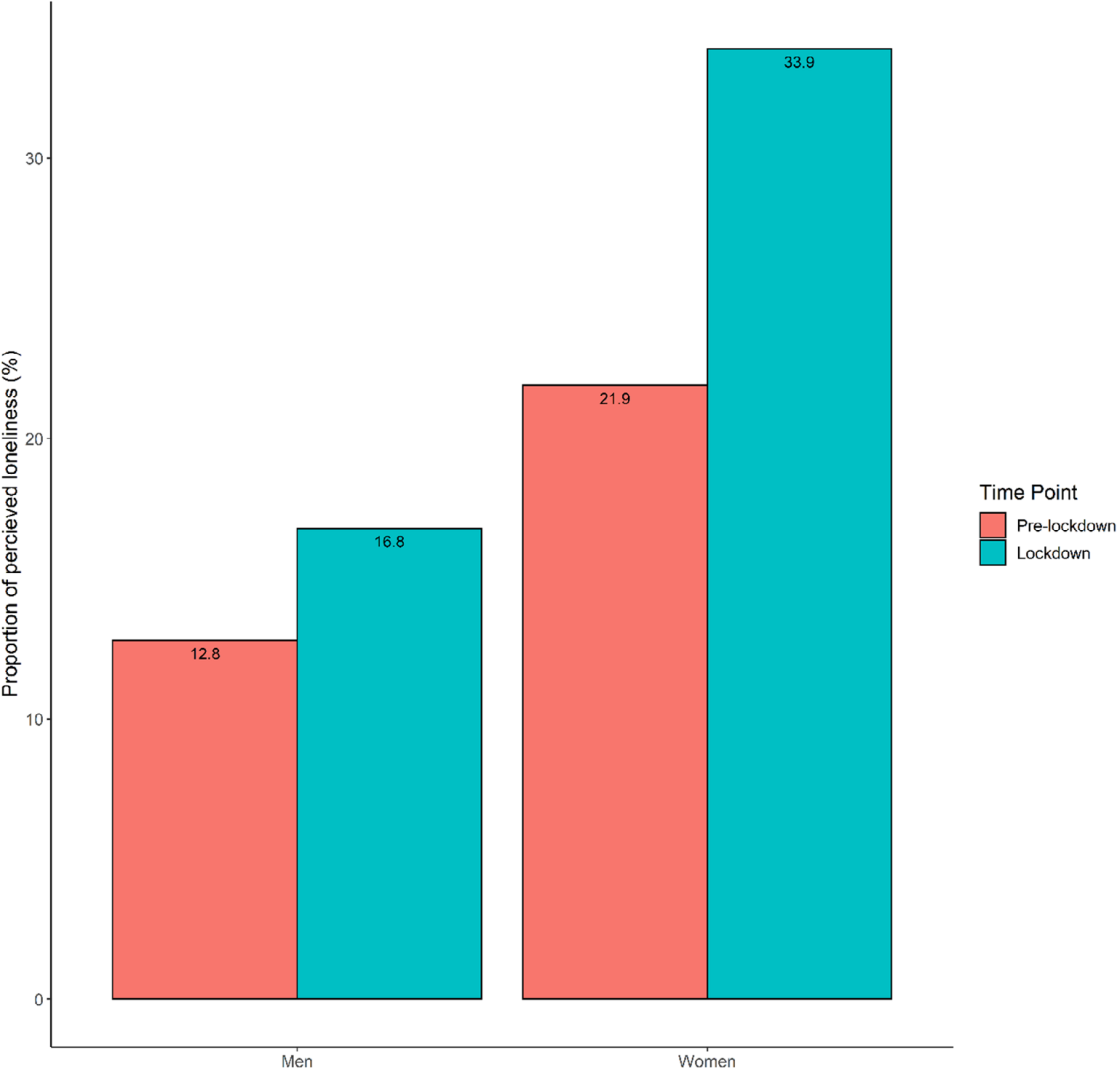
Prevalence of perceived loneliness in men and women before (wave 3) and during the pandemic (OPPO).

Table 2 & Figure 2 shows participants' perceived loneliness before and during the COVID‐19 pandemic. Before the pandemic prevalence of loneliness in older people was measured at 17.2%, whereas after the introduction of social distancing during the pandemic loneliness increased to 25.0%, a statistically significant increase of 7.8 percentage points (*p ≤* 0.01).

**TABLE 2:** Descriptive statistics for social contact and perceived loneliness before (wave 3) and during the pandemic (OPPO).

		Pre‐pandemic		During pandemic	
		*N*	%	*N*	%
Seeing relatives in person	Monthly or less	121	31.9	‐	‐
	At least weekly	183	48.3	‐	‐
	Daily	75	19.8	‐	‐
Attending community, religious or social groups	No	153	40.4	‐	‐
	Yes, less than weekly	70	18.5	‐	‐
	Yes, weekly or more	156	41.2	‐	‐
Speaking to friends or relatives via phone/video	Monthly or less	90	23.7	43	11.4
	At least weekly	207	54.6	173	45.7
	Daily	82	21.6	163	43.0
Separation from friends and family during the pandemic	No	‐	‐	159	42.0
	Yes	‐	‐	220	58.1
Met with friends/family outside the house in the past month	No	‐	‐	213	56.2
	Yes	‐	‐	166	43.8
Self‐quarantined in the past month	No	‐	‐	199	52.5
	Yes	‐	‐	180	47.5
Loneliness	No	314	82.9	284	74.9
	Yes	65	17.2	95	25.0

### Social contact

Table 2 shows descriptive statistics for face‐to‐face and telephone contact before and during the pandemic. Before the pandemic, over two thirds (68.1%) of participants had seen relatives at least weekly, and two in five (41.2%) attended in‐person meetings or groups weekly or more. One in five (21.6%) spoke to friends or relatives daily on the telephone, and more than half at least weekly (54.6%). Almost a quarter of participants had no telephone contact for at least a month at a time.

During the pandemic, the proportion of people speaking to friends and relatives on the telephone daily doubled (43.0%, *p* < 0.01), with at least weekly communication remaining high (45.7%, *p* < 0.01). During the pandemic, the proportion of people communicating via phone/video overall increased, although there remained a small proportion who had monthly, less or possibly no contact during the pandemic (11.4%).

Inevitably during the pandemic restrictions, many experienced separations from family and friends (58.1%), and most reported not being able to meet outside of their house in the past month (56.2%). Almost half of the sample reported having self‐quarantined in the past month (47.5%).

### Change in loneliness

Table 3 shows the results of both univariable and multivariable logistic regression modeling for risk of increased perceived loneliness.

**TABLE 3:** Univariable and multivariable regression models for risk of perceived loneliness during the pandemic (OPPO).

	Univariable		Multivariable	
	OR	95% CI	OR	95% CI
Demographic				
Living alone				
No				
Yes	1.8	1.3–2.3	2.9	1.5–5.5
Sex				
Male				
Female	2.5	1.6–4.1	1.8	0.9–3.3
Self‐assessed health				
Excellent/good				
Fair/poor	1.8	1.1–2.9	1.8	1.0–3.2
Interview date				
8 June–3 July				
4 July–31 July	1.6	0.7–3.5	1.3	0.5–3.5
1 August–13 September	0.7	0.3–1.5	0.4	0.1–1.1
14 September–26 October	1.5	0.7–3.3	0.8	0.3–2.1
Social contact – pre‐pandemic				
Pre‐pandemic attendance at community, religious or social groups				
No				
Yes, less than weekly	0.9	0.5–2.0	0.7	0.3–1.8
Yes, weekly or more	2.1	1.2–3.5	2.3	1.2–4.4
Social contact – during the pandemic				
Separation from friends and family during the pandemic				
No				
Yes	1.8	1.1–3.0	1.9	1.0–3.6
During‐pandemic telephone contact				
Monthly or less				
At least weekly	0.5	0.2–1.0	0.4	0.1–1.0
Daily	1.1	0.5–2.2	0.6	0.2–1.6
Prior loneliness				
No				
Yes	16.0	8.5–30.2	13.2	6.2–27.2

Prior loneliness was the strongest predictor of loneliness during the pandemic, with those who were lonely at the time of the Wave 3 interviews having more than 12 times higher odds of being lonely during the pandemic (OR: 13.2, 95% CI: 6.24–27.18).

Of the demographic factors assessed, living alone was shown to have the most considerable effect on perceived loneliness (OR: 2.9, 95% CI: 1.5–5.5). Women had higher odds of experiencing loneliness during the pandemic (OR: 1.8, 95% CI: 0.9–3.3), as did those self‐rating their health as fair or poor for their age (OR 1.8, 95% CI 1.0–3.3). Those interviewed between 1 August and 13 September, the time of most minor restrictions on social contact, had lower odds of loneliness than those interviewed in the first period (OR 0.4 95% CI 0.1–1.1).

Of the pre‐pandemic measures, attending community, religious or social groups weekly or more was predictive of loneliness during the pandemic (OR: 2.3, 95% CI: 1.2–4.4). For during‐pandemic social contact, being separated from friends and family was associated with a twofold increase in odds of loneliness (OR: 1.9, 95% CI: 1.0–3.61). Daily (OR: 0.6, 95% CI: 0.2–1.6) or weekly phone contact was associated with reduced odds of loneliness compared to monthly or less (OR: 0.4, 95% CI: 0.14–1.00).

## DISCUSSION

### Summary of findings

One in four people in the sample reported loneliness (25.1%) compared with just over one in six people the previous year (17%). Prior feelings of loneliness maintained the strongest association with loneliness during the pandemic, albeit with low precision. In terms of demographic and social factors, living alone was associated with 2.7 times increase in loneliness; more than double the odds of loneliness was also found in participants who reported regular social interactions (weekly or more attendance at groups or events) before the pandemic.

#### Changes in loneliness in older people during the COVID‐19 pandemic

This study first addressed whether the COVID‐19 pandemic is associated with the increased prevalence of loneliness among older people. It found that just over one in six older adults were lonely before the pandemic, rising to just over one in four during the pandemic. This finding is similar to the results of some other repeated measures or longitudinal studies of older people (typically 60 and over), which also found an increased prevalence of loneliness during the pandemic period.^22^, ^26^, ^27^, ^28^, ^29^, ^49^ This study adds to previous literature by confirming a similar pattern at an older age range. Increased loneliness is also consistent with what would be expected from the literature on the impact of life events that disrupt social contact.^48^

The prevalence of loneliness before the pandemic was lower in this sample than in other UK population‐based studies.^50^, ^51^ The during‐pandemic prevalence is also lower than in the same pre‐pandemic studies^50^, ^51^ and several during‐pandemic studies conducted in the UK and elsewhere.^31^, ^49^, ^52^ However, it is comparable with the during‐pandemic prevalence of two UK cross‐sectional studies.^53^, ^54^ Although an increase has been observed and is of concern given the health consequences of loneliness, the absolute prevalence in this sample has not reached unheralded levels compared to some UK pre‐pandemic estimates. One could see this as evidence that the pandemic is not triggering the unprecedented rates of loneliness that were feared, suggesting greater resilience among older people than expected.^20^, ^21^ Alternatively, it could be argued that we have been experiencing an ‘epidemic’ of loneliness for some time; hence the prevalence estimates seen here are not out substantially higher than typical loneliness estimates, despite the impact of the pandemic.

### Strengths and limitations

A key strength of this study is the use of data on experiences of the COVID‐19 pandemic embedded within an established longitudinal study. The study sample was drawn from an initial population‐representative sample, albeit with substantial attrition. Detailed data from before and during the pandemic were available, including risk and protective factors rarely explored in the existing literature. Data quality was good, with relatively low levels of missing data.

The sample was drawn from an initially population‐representative sample but with substantial attrition since initial recruitment in 2008–2011. Attrition, whether through dropout or survival, is associated with variables known to be associated with loneliness, such as female gender, health and deprivation^37^ and thus may have led to an under‐estimate of loneliness prevalence. These are challenges common to many longitudinal studies of aging.^55^, ^56^ The recruitment process for this study from the initial sample also excluded those with memory impairment, which again could lead to underestimation of loneliness.^5^

The sample comprised individuals aged 75 years and over, and more than half were over 80 years. This contrasts with most existing research literature which typically focuses on those aged 60 years (6, 7)or 65 years and above.^8^, ^9^ The study therefore adds to understanding impacts of COVID‐19 policies on loneliness in older age ranges. The older age group might be expected to have a higher prevalence of pre‐pandemic loneliness^10^; however, this was not borne out in this sample. Prior work also suggests that decreased social contact has a larger effect on persons in the older age group.^10^ The change from pre‐ to during‐pandemic levels of loneliness may therefore be greater in our sample than for the general older population.

The difficulty in accurately measuring loneliness is well‐recognized.^22^ In this study, validated direct measures of loneliness were used.^49^ However, using a direct measure in the absence of an accompanying indirect measure may have led to some under‐reporting of the outcome.^50^ Other studies have also found that men have higher under‐reporting rates than women, meaning that ascertainment bias may be present in the association between women and loneliness identified in this study, potentially overestimating the strength of this association.^57^

Different direct measures of loneliness were used in the pre‐and during‐pandemic questionnaires, giving rise to whether the observed result could be an artifact of the different approaches to measurement. However, sensitivity analysis suggested that a relatively high (36%) misclassification rate of new cases of loneliness would be required for this to be a spurious finding.

## CONCLUSION

This research highlights an increase in loneliness in older people during the COVID‐19 restrictions, including a potentially higher‐risk group comprising those who had previously been socially active through community and religious groups. These individuals may need to be supported to rebuild links with those groups, whether that is through resuming face‐to‐face contact as restrictions ease, or through technology‐mediated contact.

## AUTHOR CONTRIBUTIONS

Connor D Richardson and Hannah Roscoe were responsible for writing of the manuscript and data analysis. Emma Green was responsible for data management of CFAS and OPPO. Racheal Brooks & Linda Barnes were responsible for project management in CFAS and OPPO. Carol Brayne & Fiona E Matthews were responsible for overall supervision of the study. All authors contributed to the final draft of the manuscript.

Connor D Richardson prepared and submitted the manuscript. The work in this paper originated from Hannah Roscoe's MSc dissertation.

## CONFLICT OF INTEREST

The authors declare that there is no conflict of interest.

## SPONSOR'S ROLE

CFAS II Wave 3 was funded by ARUK. Following the onset of the COVID‐19 pandemic, OPPO Wave 1 was funded by ARUK, OPPO Wave 2 was funded by UKRI and ESRC.

## Supporting information


**Figure S1.** Full CFAS II & OPPO data flow.
**Figure S2.** Timeline of COVID‐19 restrictions and OPPO interviews, March November 2020.
**Table S1.** Overview of COVID‐19 restrictions January to November 2020.
