# Supplementary material for: Impact of COVID‐19 policies on perceptions of loneliness in people aged 75 years and over in the cognitive function and aging study (CFAS II)
Source: J Am Geriatr Soc. 2022 Nov 12:10.1111/jgs.18099. Online ahead of print. doi: 10.1111/jgs.18099 (PMC9877735; doi:10.1111/jgs.18099)
Supplement: Supplementary file 1 — Figure S1. Full CFAS II & OPPO data flow. Figure S2. Timeline of COVID‐19 restrictions and OPPO interviews, March November 2020. Table S1. Overview of COVID‐19 restrictions January to November 2020. [file JGS-9999-0-s001.pdf]

Supplementary Material

Supplementary figure 1: Full CFAS II & OPPO data flow

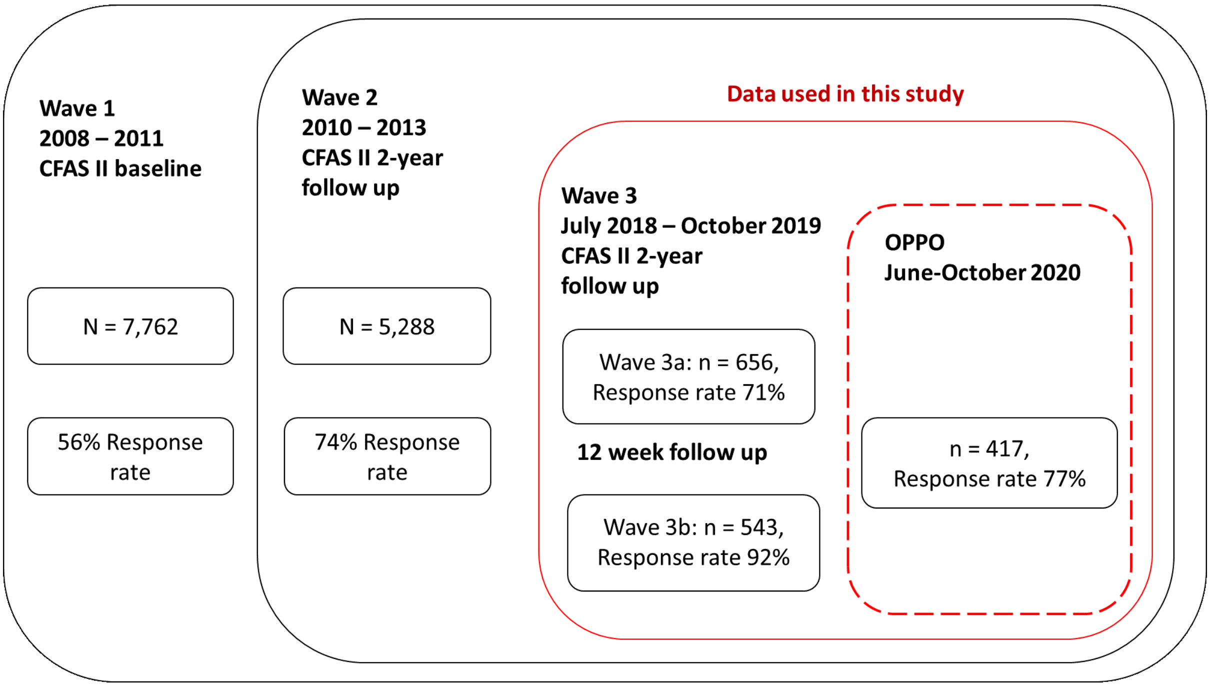

Supplementary figure 2: Timeline of COVID-19 restrictions and OPPO interviews, March to November 2020

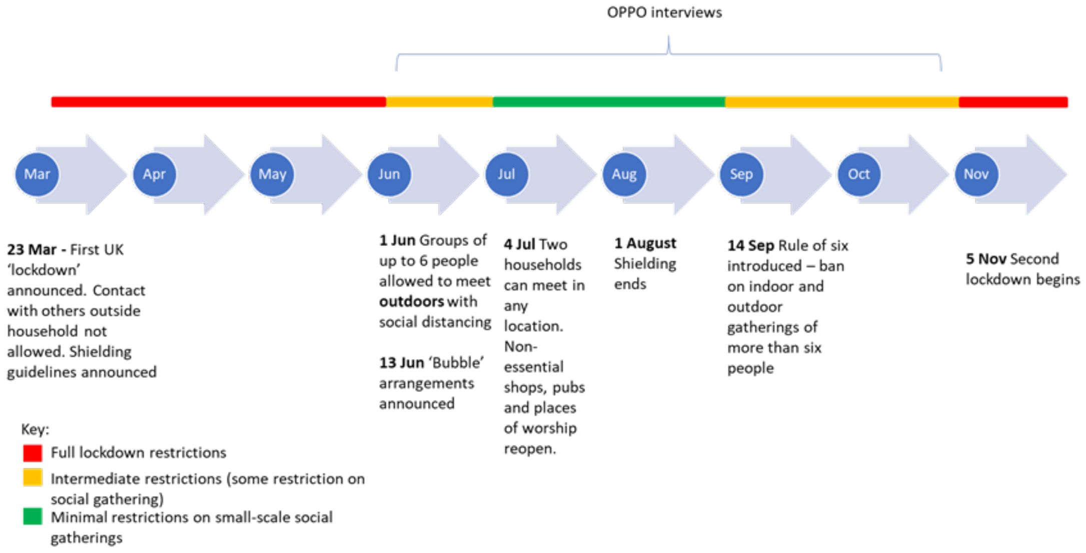

Supplementary table 1: Overview of COVID-19 restrictions January to November 2020

23 January	First COVID-19 case enters UK
5 March	Coronavirus listed as a notifiable disease in the UK
16 March	Government announces new social distancing measures. Those over 70 advised to self-isolate.
**23 March**	**First UK ‘lockdown’ announced and shielding measures introduced**
1 June	Groups of up to six people allowed to meet outdoors
13 June	‘Bubble’ scheme announced, enabling single people to link with another household
15 June	Non-essential shops and places of worship reopen in England
**4 July**	**Restrictions eased, two households can meet in any location**
**1 August**	**Shielding guidance paused**
14 August	Lockdown restrictions eased further, including reopening of theatres
14 September	‘Rule of six’ introduced – ban on indoor and outdoor gatherings of more than six people
**5 November**	**Second lockdown begins**
